# The European landscape for gene therapies in orphan diseases: 6-year experience with the EMA Committee for Orphan Medicinal Products

**DOI:** 10.1016/j.ymthe.2023.09.020

**Published:** 2023-10-04

**Authors:** Gloria M. Palomo, Tomas Pose-Boirazian, Frauke Naumann-Winter, Enrico Costa, Dinah M. Duarte, Maria E. Kalland, Eva Malikova, Darius Matusevicius, Dinko Vitezic, Kristina Larsson, Armando Magrelli, Violeta Stoyanova-Beninska, Segundo Mariz

**Affiliations:** 1 Agencia Española de Medicamentos y Productos Sanitarios, Calle Campezo 1 Edificio 8, 28022 Madrid, Spain; 2 Orphan Medicines Office, European Medicines Agency, Domenico Scarlattilaan 6, 1083 HS Amsterdam, the Netherlands; 3 Bundesinstitut für Arzneimittel und Medizinprodukte, Kurt-Georg-Kiesinger-Allee 3, 53175 Bonn, Germany; 4 Agenzia Italiana del Farmaco, Via del Tritone 181, 00187 Rome, Italy; 5 INFARMED - National Authority of Medicines and Health Products, I.P., Avenida do Brasil 53, 1749-004 Lisbon, Portugal; 6 Universidade de Lisboa, Faculdade de Farmácia, Avenida Professor Gama Pinto, 1649-003 Lisbon, Portugal; 7 Statens Legemiddelverk/The Norwegian Medicines Agency, Grensesvingen 26, 0663 Oslo, Norway; 8 State Institute for Drug Control, Kvetná 11, 825 08 Bratislava, Slovakia; 9 Department of Pharmacology and Toxicology, Comenius University, Odbojárov 10, 832 32 Bratislava, Slovakia; 10 Läkemedelsverket, Dag Hammarskjölds väg 42, 75237 Uppsala, Sweden; 11 Rijeka University School of Medicine and University Hospital Centre Rijeka, Braće Branchetta 20, 51000 Rijeka, Croatia; 12 National Center for Drug Research and Evaluation, Istituto Superiore di Sanità, Viale Regina Elena 299, 00161 Rome, Italy; 13 College ter Beoordeling van Geneesmiddelen, Graadt van Roggenweg 500, 3531 AH Utrecht, the Netherlands; 14 Committee for Orphan Medicinal Products, European Medicines Agency, Domenico Scarlattilaan 6, 1083 HS Amsterdam, the Netherlands

**Keywords:** gene therapy, orphan medicinal product, viral vector-based medicines, COMP, regulatory incentives, rare diseases, EMA, PRIME, prevalence, orphan designation

## Abstract

In 2000, the European Union (EU) introduced the orphan pharmaceutical legislation to incentivize the development of medicinal products for rare diseases. The Committee for Orphan Medicinal Products (COMP), the European Medicines Agency committee responsible for evaluation of applications for orphan designation (OD), received an increasing flow of applications in the field of gene therapies over the last years. Here, the COMP has conducted a descriptive analysis of applications regarding gene therapies in non-oncological rare diseases, with respect to (a) targeted conditions and their rarity, (b) characteristics of the gene therapy products proposed for OD, with a focus on the type of vector used, and (c) regulatory aspects pertaining to the type of sponsor and development, by examining the use of available frameworks offered in the EU such as protocol assistance and PRIME. It was noted that gene therapies are being developed by sponsors from different backgrounds. Most conditions being targeted are monogenic, the most common being lysosomal disorders, and with a very low prevalence. Generally, adeno-associated viral vectors were being used to deliver the transgene. Finally, sponsors are not frequently using the incentives that may support the development and the reasons for this are unclear.

## Graphical abstract

**Figure:**
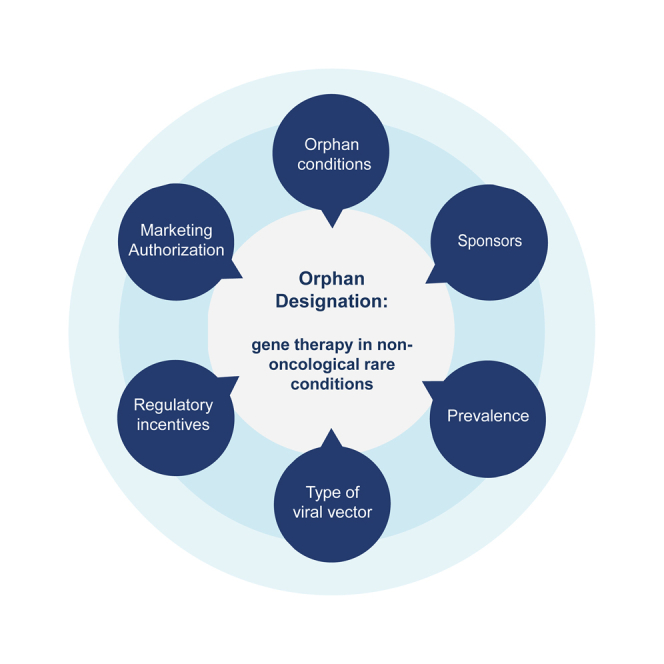

## Teaser

Palomo, Mariz and colleagues describe the particularities of viral vector-based gene therapy products seeking for the OD in rare non-oncological conditions. Moreover, the authors dig into the usage of regulatory incentives and how the sponsors can benefit from these schemes.

## Introduction

Over the past two decades, there has been an increasing interest in developing advanced therapy medicinal products (ATMPs) that have been subject of evaluation under different regulatory procedures since the introduction of their dedicated framework (Regulation (EC) No 1394/2007). However, the Committee for Orphan Medicinal Products (COMP) received their first orphan designation (OD) application for a gene therapy product as early as 2001; throughout the years, ODs for gene therapies have subsequently been submitted under the orphan framework (Regulation (EC) No 141/2000). In that regard, a noticeable increase in this type of products has been seen by the COMP in the last decade.^1^

Many OD applications for ATMPs pertain to rare genetic disorders and oncology. The National Human Genome Research Institute defines genetic disorders as “a disease caused in whole or in part by a change in the DNA sequence away from the normal sequence.” It has recently been reported that more than one-half of the clinical trials in this group focused on metabolic, eye and blood coagulation disorders.^2^

Viral-derived vectors are the most common tool used to deliver foreign genetic material into eukaryotic cells, mainly because of their well-known efficacy and safety profile.^3^ Over the past decade, several gene therapy medicines have received regulatory approval for the treatment of different diseases and an increase in the number of these medicines is expected in the upcoming years, as seen in the current clinical landscape.^4^

Based on the setting of genetic intervention, gene therapies are commonly referred to as *ex vivo*, *in vivo*, or *in situ* (*in vivo* local delivery). *Ex vivo* (also called *in vitro*) gene therapies involve the use of target cells removed from the patient’s body that are genetically engineered to allow for the desired phenotypic correction. This is followed by reintroduction of the engineered cells into the patient. In the case of *in vivo* therapies, the therapeutic vector is administered systematically as in the blood circulation or using alternative routes targeting different anatomical compartments of the patient (in case of using an *in situ* approach), and it is expected to enter certain types of cells where it exerts a therapeutic effect via genetic manipulation.

In this descriptive analysis, we sought to review the applications of gene therapies, viral vector based, seeking for the orphan drug designation in the European Union (EU) in the period 2016–2021, for the treatment of rare non-oncological conditions. Analysis of the whole package of OD for viral-vector-based gene therapy products showed that the majority of the designations are intended for non-oncological conditions; very few are targeting rare cancer conditions, with the exception of chimeric antigen receptor (CAR)-T cells and similar products, which will be subject of a different analysis due to the inherent differences ascribed to the type of product. Only four ODs were granted for replicating viral vectors (adenoviral, lentiviral, and retroviral vectors) in oncological indications. This is further supported by the fact that only one replicating viral vector received a license, which was Imlygic, the first oncolytic immunotherapy, for the treatment of melanoma in 2015. Since then, no further marketing authorization applications (MAAs) have been received for this type of product via the centralized procedure in Europe. As mentioned, ODs for CAR-T cells will be the subject of another publication.

OD offers incentives both in the pre-licensing stage as well as licensing and post-licensing to the designation holder. The Orphan Regulation offers protocol assistance (PA), which is a type of scientific advice dedicated for orphan medicines for rare diseases, during the research and development phase of a designated product.^5^ PA involves all aspects of product development (quality, non-clinical and clinical issues, together with methodological and overall development strategy aspects) in addition to specific questions addressed by the COMP. A sponsor with an OD can come as often as they need, and the framework offers fee reductions based on the type of sponsor submitting. Additionally, fee reductions can be obtained for orphan designated products at the licensing stage. Once a designation holder is successful in obtaining a license for their product and, upon revision of the criteria as set up in the legislation, maintains the orphan status, the medicine in the orphan condition is eligible for a 10-year market exclusivity. If the marketing authorization (MA) holder has a compliant completed pediatric investigation plan, then the medicine is eligible for an additional 2-year period of market exclusivity, leading to a maximum of 12 years per product and condition.^6^

Regarding PRIority MEdicines (PRIME), this scheme was launched by the European Medicines Agency (EMA) to enhance support for the development of medicines that target an unmet medical need.^7^ This voluntary scheme is based on enhanced interaction and early dialogue with developers of promising medicines, to optimize development plans and speed up evaluation so these medicines can reach patients earlier. To be accepted for PRIME, a medicine must demonstrate the potential to address an unmet medical need to a significant extent. This could mean, for example, introducing new methods of therapy or improving existing ones. To justify such potential, applicants must provide any available data showing a meaningful improvement of clinical outcomes, that show the treatment can prevent or delay the onset, as well as reduce the duration of a given condition, or show an improvement in the morbidity or mortality. Applicants from academia and small to medium enterprises (SMEs), who generally have less experience of the regulatory landscape, may submit an eligibility request for Early Entry PRIME status if compelling non-clinical *in vivo* data in a relevant model provide early evidence of promising activity, or proof-of-principle, and first-in-human studies indicate adequate exposure for the desired pharmacotherapeutic effects and tolerability.^8^

As non-oncological rare diseases seem to be the main target of viral-vector-based gene therapies for OD and the associated incentives, the aim of this paper is to provide insights on the regulatory decision-making process for these products, with a view that our findings will ultimately help to improve the successful development. In addition to the classification of the applications in terms of therapeutic group and prevalence, we investigated the most common type of viral vector used over the years and how the medicines were delivered to the patients' cells.

## Results

### Regulatory classification

One hundred fourteen applications were examined. Most were accepted at their first application and only one sponsor withdrew the application during the period examined. This dossier was submitted 2 years later and resulted in a positive opinion from the COMP. Since this dossier was submitted twice, we have included both applications in the analysis. There were no negative opinions for the period covered.

**Figure 1:**
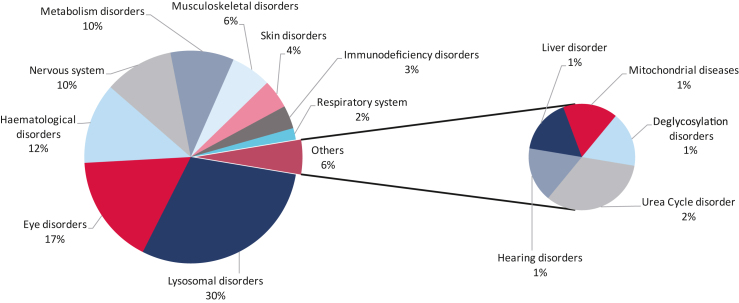
Distribution of conditions for viral vector-based gene therapy products submitted for orphan medicinal product designation Analysis of the proportion of applications per therapeutic area. The total number of OD applications was 114.

The respective orphan conditions were classified into therapeutic areas following Medical Dictionary for Regulatory Activities (MedDRA) terms. The most common therapeutic area targeted were lysosomal disorders representing approximately one-third (30%) of all the submissions that were assessed by the COMP. There was an even spread between eye, hematological, metabolic, and nervous system disorders (between 17% and 10%). All other conditions represented a small proportion of the submissions (Figure 1). Individual conditions by therapeutic area are presented in Table 1. In this sense, retinitis pigmentosa (with eight applications), followed by neuronal ceroid lipofuscinosis and hemophilia A, with six applications each, were the orphan conditions most represented in our analysis.

**Table 1:** Orphan conditions as seen in products submitted for orphan medicinal product designation for the period 2016–2021

Lysosomal disorders	Eye disorders	Hematological disorders
Neuronal ceroid lipofuscinosis, Fabry disease, glycogen storage disease type II (Pompe’s disease), Gaucher disease, mucopolysaccharidosis type I, mucopolysaccharidosis type II (Hunter’s syndrome), GM1 gangliosidosis, GM2 gangliosidosis, Krabbe disease, Canavan disease, cystinosis, metachromatic leukodystrophy, mucopolysaccharidosis type IIIB (Sanfilippo B syndrome), mucopolysaccharidosis type IIIA (Sanfilippo A syndrome), mucopolysaccharidosis type IVA (Morquio A syndrome)	Retinitis pigmentosa, Leber’s congenital amaurosis, inherited retinal dystrophies, achromatopsia caused by mutations in the CNGA3 gene, cone-rod dystrophy, RDH12 mutation associated retinal dystrophy, Stargardt’s disease, achromatopsia, Retinitis pigmentosa caused by mutations in the RPGR gene	Hemophilia A, hemophilia B, Diamond-Blackfan anemia, beta thalassemia intermedia and major, sickle cell disease
**Nervous system**	**Metabolic disorders**	**Musculoskeletal disorders**
Frontotemporal dementia, Angelman syndrome, Friedreich’s ataxia, Rett syndrome, Huntington’s disease, megalencephalic leukoencephalopathy with subcortical cysts, SLC13A5-epileptic encephalopathy deficiencies	Wilson’s disease, phenylalanine hydroxylase deficiency, aromatic L-amino acid decarboxylase deficiency, aspartylglucosaminuria, congenital adrenal hyperplasia, Crigler-Najjar syndrome, glycogen storage disease type Ia, methylmalonic acidemia	Duchenne muscular dystrophy, limb-girdle muscular dystrophy, oculopharyngeal muscular dystrophy osteopetrosis, spinal muscular atrophy
**Skin disorders**	**Immunodeficiency disorders**	**Respiratory system**
Epidermolysis bullosa, autosomal recessive congenital ichthyosis	X-linked severe combined immunodeficiency, adenosine deaminase 2 deficiency, leukocyte adhesion deficiency type I	Cystic fibrosis
**Urea cycle disorder**	**Hearing disorders**	**Liver disorder**
Ornithine transcarbamylase deficiency	Otoferlin gene-mediated hearing loss	Progressive familial intrahepatic cholestasis
**Mitochondrial diseases**	**Deglycosylation disorders**	
Leigh syndrome	NGLY1 deficiency	

**Figure 2:**
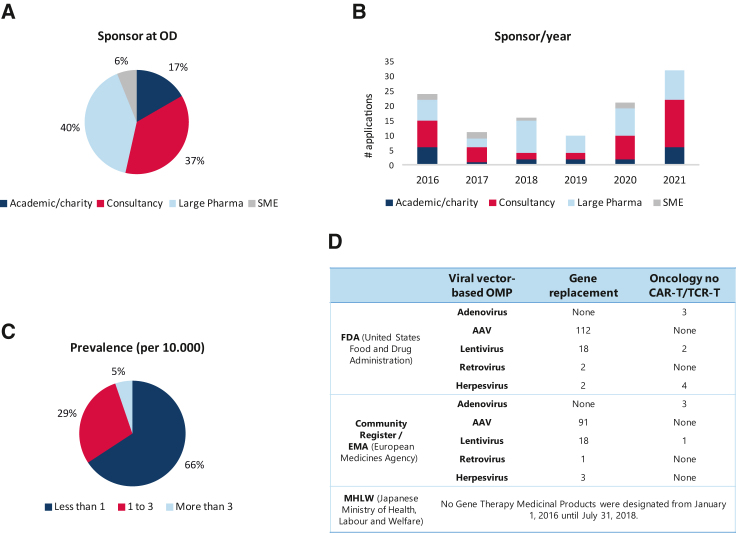
Characteristics of the orphan medicinal products in EU and in other regions (A) The sponsor at the time of initial OD was classified in 4 different categories. Data shown as percentage. (B) Distribution of the type of sponsor per year at the time of initial OD. (C) Proportion of OD applications categorized according to the prevalence of the orphan condition. (D) Comparative table showing viral vector-based orphan designated products, as shown in the Community Register (after COMP/EMA opinion) and in the FDA (for the United States), for the period 2016–2021. No gene therapy medicinal products obtained OD in Japan, from January 1, 2016, until July 31, 2018 (no updates are available after this date).

With regard to the type of sponsor, large pharmaceutical companies were the most common requestors of OD submissions (40%), closely followed by consultancy agencies. Academics/charities represented 17% of the submissions (Figure 2A). SME only accounted for 6% of all the submissions. When the sponsors were analyzed per year (Figure 2B), we found that the applications from consultancy decreased substantially for 2018 and 2019, showing a recovery in the following years. Interestingly, this decrease was not evident for OD submissions from large pharmaceutical companies.

When the prevalence of the orphan condition was studied, it was revealed that the largest number of submissions (66%) had a prevalence below 1 in 10,000 and those above 3 in 10,000 represented only 5% of the total (Figure 2C).

The number of gene therapy ODs, the type of viral vector used, and the orphan condition or indication were studied in an international scenario, for those regions in which a specific framework to foster the development of orphan medicinal products is in place. For this purpose, a comparison between designations granted by the European Commission (through the COMP), by the U.S. Food and Drug Administration (FDA) and by MLHW (Japanese Ministry of Health, Labour and Welfare, through their agency PMDA), are shown in Figure 2D.

There were 143 gene therapies submitted to the FDA, of which 134 were for non-oncological conditions. Of these 118 were for adeno-associated viral (AAV) vectors, 18 were for lentiviral vectors and only 2 for retroviral vectors and other 2 for herpes viral vectors. In oncological indications (this excluded CAR-T cells and similar types of products), three were for adenoviral vectors, two were for lentiviral vectors, and four for herpes viral vectors. In comparison, in Europe there was a total of 117 designations, with 113 intended for non-oncological conditions and with only 4 for oncological rare diseases (excluding again the CAR-T cell products). Of the later, three products were based in adenoviral vectors and only one was a retroviral vector. No submissions of gene therapies were reported on the publicly reported list on the Japanese Pharmaceuticals and Medical Devices Agency (PMDA) website, with the caveat that the data available was between 2016 and 2018.

### Product specifics classification

**Figure 3:**
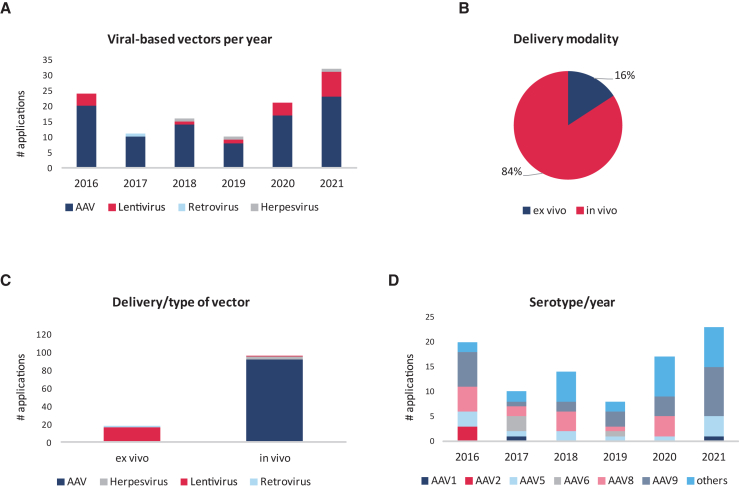
In-depth analysis of the products assessed by the COMP (A) Distribution per year of viral-based vectors used for gene replacement purposes in OD applications between 2016 and 2021. (B) Proportion of OD applications regarding the treatment modality (*in vivo*/*ex vivo*). (C) Number of viral-based vectors used per treatment modality. (D) AAV serotype distribution per year. The term “others” encompass novel serotypes such as serotype 2.5T, 2.7m8, 3B, Anc80, HSC15, hu37, hu68, LK03, PTC3, rh10, rh74, and S3.

Following a detailed year-by-year analysis for the whole period covered, the most frequently submitted viral vector-based therapies were the ones utilizing AAV vectors adding up to a total of 92 applications for the 6 years examined, followed to a much lesser degree by the lentivirus (18 submissions). Very few retroviral and herpesvirus-based vectors were submitted (Figure 3A). When the applications are classified according to the modality of treatment, the majority (84%) are accounted as *in vivo* delivery (including *in situ*), with *ex vivo* delivery systems accounting for the rest (16%) (Figure 3B). Regarding the type of viral vector used for *in vivo* delivery, all the AAVs and herpesvirus-based products cluster into this category and only one lentivirus was intended for *in vivo* delivery (a product for hemophilia B). In contrast, the *ex vivo* products all are based on integrative vectors like lentivirus and, exceptionally, one retroviral vector (a product for epidermolysis bullosa) (Figure 3C).

Given the observed preference for AAV-derived vectors, we studied the serotypes in terms of classical serotypes (AAV1–13) and new isolated or recombinant serotypes. In our analysis, there is a trend toward increased use of new serotypes, such as rh10, rh74, and LK03, among others, for the last years of the analysis (Figure 3D).

### Use of regulatory incentives

**Figure 4:**
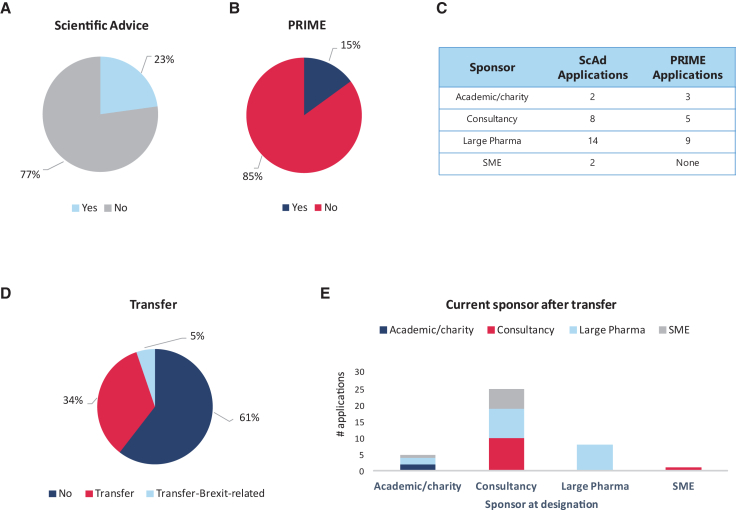
Use of regulatory incentives (A) Proportion of orphan designated medicines which have received PA during the product development. (B) Proportion of orphan medicinal products that have been included in the PRIME scheme. (C) Comparative table of the number of scientific advice and PRIME applications per type of sponsor. (D) Percentage of transfers of the initial OD. Some transfers could be ascribed to Brexit and are shown as a separate category. (E) Distribution of the transfers of the ODs according to the source and destination.

The number of PA completed was 26 of 114 (23%) of successful granted ODs (Figure 4A). When this number is split into the different types of sponsors, large pharma holds the greater share of submissions (14 in total), followed by consultancies (eight applications) and SME and academia with two applications for PA each (Figure 4C).

PRIME designations of gene therapies with a granted OD was 15% (or 17 out of 114 products in total) (Figures 4B and 4C). Submissions for PRIME showed that large pharma was the most common applicant (nine submissions), followed by consultancies (five applications), and academia/charity (only three applications). There were no applications from SMEs.

Transfers between sponsors were also recorded (Figures 4D and 4E). Sixty-one percent of designations did not have a transfer. Thirty-four percent of the total ODs underwent transfers, while 5% were transfers associated with the Brexit (from the same sponsor, registered in the UK to any other UE region). Most transfers occur from consultancies to other consultancies or large pharma or SMEs. All the transfers originating in large pharma had the same type of sponsor as destination and, in contrast, although few transfers occur from academics (only five), those were destined to large pharma, academia and SME. There was only one transfer in between SMEs.

### Gene therapies authorized in the EU

The OD and PRIME status of the authorized gene therapies were studied upon consultation in relevant registries (Table 2). Overall, eleven gene therapies received a MA in the EU. All gene therapies except for one (Imlygic) had OD, given that melanoma is not an orphan condition. Glybera, Zynteglo, and Skysona withdrew their MAs for commercial reasons (detailed withdrawal statements can be found on EMA website). At the same time, those medicines were removed from the Community Register of orphan medicinal products. Five products received PRIME designation: Zynteglo, Zolgensma, Skysona, Roctavian, and Hemgenix. These products received a MA after 2016, when the PRIME scheme was already introduced in the EU (which occurred in 2016).

**Table 2:** Overview of the gene therapy medicinal products authorized in the EU

Gene therapy medicinal products				
Product	Orphan condition	Orphan status/PRIME status at MAA	MA applicant	MA status
Glybera	lipoprotein lipase deficiency	OMP	uniQure biopharma B.V.	withdrawn
Imlygic	*melanoma is not an orphan condition*	N/A	Amgen Europe B.V.	authorized
Strimvelis	severe combined immunodeficiency due to adenosine deaminase deficiency	OMP	Orchard Therapeutics (Netherlands) B.V.	authorized
Luxturna	inherited retinal dystrophies/retinitis pigmentosa	OMP	Novartis Europharm Limited	authorized
	inherited retinal dystrophies/Leber’s congenital amaurosis	OMP	Novartis Europharm Limited	authorized
Zynteglo	beta-thalassemia intermedia and major	OMP and PRIME	bluebird bio (Netherlands) B.V.	withdrawn
Zolgensma	spinal muscular atrophy	OMP and PRIME	Novartis Europharm Limited	authorized
Libmeldy	metachromatic leukodystrophy	OMP	Orchard Therapeutics (Netherlands) B.V.	authorized
Skysona	adrenoleukodystrophy	OMP and PRIME	bluebird bio (Netherlands) B.V.	withdrawn
Upstaza	aromatic L-amino acid decarboxylase deficiency	OMP	PTC Therapeutics International Limited	authorized
Roctavian	hemophilia A	OMP and PRIME	BioMarin International Limited	authorized
Hemgenix	hemophilia B	OMP and PRIME	CSL Behring GmbH	authorized

N/A, not assessed; OMP, orphan medicinal product.

A deeper analysis revealed that Strimvelis and Libmeldy were originally designated to a charity, Telethon Italy. Then designations were transferred first to Glaxo Smith Kline and latter to Orchard Pharmaceuticals, which is the final MA applicant for both products. Consultants were used in two cases, Luxturna and Upstaza. Both these products originated in the United States. Luxturna was designated to Alan Boyd Consultants who represented Spark Therapeutics. Afterward, Spark Therapeutics transferred their designation and product to Novartis. Upstaza was designated to Voisin Consultants, who represented Agilis Biotherapeutics, who then transferred the designation to PTC Therapeutics Europe. Glybera and Zolgensma both obtained their original ODs through SMEs. Glybera was transferred to UniQure B.V. and Zolgensma to Novartis. Roctavian, Hemgenix, Zynteglo, and Skysona all obtained their original ODs from mid-sized pharmaceutical companies.

There were no academic ODs that have obtained a market authorization.

Roctavian, Upstaza, and Hemgenix obtained their initial OD during the period covered by our analysis (2016–2021), and all of them received PA during their development.

## Discussion

The COMP has evaluated many applications for OD pertaining to conditions where gene therapies may be used to rectify the underlying genetic aberration and ultimately provide a long-lasting and potentially disease-modifying effect for affected patients. This potential underpins the interest in understanding the main features of the respective developments. Interestingly, as ODs are always granted for specific products in specific indications (in pairs of product/condition), our analysis yielded important observations into the product and sponsors of the OD and into the rarity and characteristics of the diseases to be treated.

A large percentage (40%) of submissions and positive designations came from large pharmaceutical companies. Consultancies represented the second largest group, making up 37% of the positive submissions. In contrast, academia/charities represented 17% of the submissions and SMEs accounted only for 6%. This distribution of sponsor differs from that described by Farkas et al., where SME accounted for more than 30% of all submissions, being the latter aligned with the general orphan figures as well.^1^^,^^9^ Those differences might be ascribed to the different pool of applications explored in Farkas et al. (all ATMPs in all types of conditions), while the current analysis is focused on viral vector-mediated gene therapy in non-oncological rare conditions, raising the question whether SME are actively involved in developing this specific type of products. As for the data aggregated under academia submissions, an increase is detected compared with the previous analysis (3.7% in Farkas et al. compared with 17% in our analysis). This might suggest that academic groups are carrying out an important share of research for these conditions and these products and, at least, they can bring their research until the milestone of OD, given that no academic ODs were granted MAs.

To better understand the observed differences, we investigated the variability in OD sponsors over the 6-year period studied. As shown in Figure 2B, large pharma are important in the submission of ODs to the COMP; however, consultancies and academics have seen a substantial growth in 2020 and 2021, after some years in which were poorly represented in the total pool. SMEs represent a small proportion of ODs; in some years, they do not appear at all. Whether there is a continuity in the clinical development and finally these products reach the stage of MA application is a question to be answered by a different analysis. Eleven gene therapies have obtained MAs in the EU through the centralized procedure (excluding CAR-T cells). Only one submission of a replicative viral vector product was a cancer therapy, namely Imlygic for the treatment of melanoma and the applicant was Amgen, a large pharmaceutical company. All others, however, show a mixed picture regarding the original sponsor for the OD with no submissions from academics reaching MA. One charity, Telethon Italy, seems to have found a pathway to get their products to patients through OD and transfer agreements to large pharma. Strimvelis and Libmeldy both originated in this charity and have obtained a license. Two ODs from SMEs achieved a license through transfers to large pharma. It was concluded that approximately one-half of the ODs which successfully obtained a MA were initially developed outside of large pharma.

As for the type of vector used, it is noted that, in the development of gene therapies, the challenge is often to find the optimal manner of delivery of a transgene in the specific tissue of interest, with a view to exert a beneficial effect relevant to the condition in question. In the data analyzed, it was noted that AAV vectors were by far the most common delivery system, used in 81% of the submissions, and were a constant through the 6-year period that was examined. There are multiple AAV serotypes described in the literature.^10^ Classical serotypes (AAV1–13) have been widely used for gene therapy purposes. However, a shift toward new serotypes, either isolated from other species or recombinant, has become more frequently used during the last years (Figure 3D). Our analysis has also shown that there is a trend toward increased use of new serotypes, such as rh10, rh74, and LK03, among others, in 2020 and 2021. Similarly, the use of AAV9 was shown to be stable or even slightly increased during 2021, probably because it is being preferentially used for targeting lysosomal disorders, which represent the biggest group of applications in our analysis.

Lentiviral vectors represented 16% of the delivery systems used and, although appearing to have waned during the interval between 2017 and 2019, they seem to have gained popularity again in 2021. Of note, Herpesviridae and Retroviridae vectors were used only marginally.

It was noted that, when compared with the ODs granted by the FDA, the similarities were striking (Figure 2D). AAV vectors were the most popular vector submitted with numbers very similar to those seen in Europe. In addition, it was also noted that, as in Europe, oncological viral vector medicines were limited in the FDA. There were no ODs for these vectors at the PMDA (presented publicly on their website up to 2018).

In a recent publication it was reported that the most common viral vector used in gene therapies in clinical trials are in fact adenoviral vectors representing approximately 50%, with AAV vectors representing 28% and lentiviral vectors accounting for 22%.^3^ In oncology, it has been reported by Urban Bezeljak that adenoviral vectors (AdVs) (in 26% of cases) are used more frequently than AAV vectors (1.6% of cases).^11^ The author also notes that replication-competent adenovirus vectors are used in oncolytic cancer therapy, while replication-incompetent AdVs are gene delivery vehicles. However, immunity toward AdVs used for gene delivery hampers their use. Regarding the use of adenovirus-derived vectors in OD, few were seen by the COMP during the years examined and this picture is reproduced when the designations granted by the FDA are reviewed (Figure 2D). This is probably ascribed to the fact that many of the conditions in which AdVs are being developed are not rare diseases.

AAV-derived vectors do not cause any human disease, are non-replicative, and have broad tissue tropism compared with the overall picture of viral-derived vectors.^11^ There has been a shift toward new AAV serotypes to address some concerns, such as immunogenicity.^10^ In our analysis, these new serotypes represent 43% of AAV applications in 2018, 47% in 2020, and 35% in 2021 (28 submissions for the whole period covered). Similar numbers were observed for AAV9, which are still widely submitted for OD to the COMP (27 applications for the 6-year period).

Prevalence was also studied and revealed that 66% of the submissions involved conditions that had a prevalence of less than 1 in 10,000. Thirty percent correspond with the intermediary prevalence of 1–3 in 10,000, and the remaining 6% addressed a prevalence of more than 3 in 10,000. When examined in the context of the prevalence of all designated orphan conditions (with 41%–46% overall pertaining to conditions affecting less than 1 in 10,000, as per the EMA Orphan Data^9^), it appears that the rarity of conditions targeted by the ATMPs is more pronounced. Hence, gene therapies using viral vector delivery systems are generally targeting very rare conditions. This is in line with the findings described by Braga et al., where the authors conducted an extensive survey among 1,430 researchers working with rare diseases from around the world on the future of genetic therapies to treat rare genetic diseases.^12^ The results from this study showed that the group “rare genetic neurological disorder” that had the highest frequency. The focus on rare diseases may have several explanations such as the fact that these are often monogenic and with few disease-causing mutations, making these disorders an “easier” target.

Examination of the rare conditions currently being targeted highlights several therapeutic areas. The COMP noted that the most frequent therapeutic area that was targeted for gene therapy at the time of initial OD were lysosomal disorders, representing 30% of submissions followed by eye (17%) and hematological (12%) disorders. Submissions for neurological and metabolic disorders represented 10% each, and the rest was a collection of other disorders, such as musculoskeletal, immunodeficiency, skin, and respiratory disorders. Individual conditions are highlighted in Table 1 for a greater understanding of the rare diseases that were targeted for OD. According to a recent pharmaceutical industry-scientific society publication, 8 of the top 10 rare diseases targeted by gene therapy are oncological except the last two, as seen all throughout 2022.^13^ In ninth place was amyotrophic lateral sclerosis (ALS) and in tenth position retinitis pigmentosa. The COMP found that they had not received any submissions for gene replacement therapies for ALS. There were however, designations granted for retinitis pigmentosa (nine submissions for our 6-year period).

Post-designation incentives are in place according to the EU legislation, such as PA, and provided by the EMA to assist and help applicants (sponsors) with an OD in their development phase. In the case of gene therapies, it was noted that only 23% of sponsors with an OD obtained PA after they received designation. In 2017, it was reported that 29.8% of ATMPs that had an OD obtained a scientific advice; most of these were gene therapies.^1^

Sponsors should, therefore, be adequately informed and encouraged to make use of this procedure to help guide them regarding potential unforeseen regulatory concerns which may affect their possibility of obtaining a MA recommendation from the EMA Committee for Medicinal Products for Human Use (CHMP). Gene therapies in rare conditions present unique challenges regarding quality concerns^14^^,^^15^ and acceptability of clinical data.^16^^,^^17^

The PRIME scheme is limited to medicines under development that are not authorized in the EU and for which the applicant intends to apply for an initial MA through the centralized procedure. The eligibility criteria for PRIME are identical to the EMA’s MAA accelerated assessment criteria, but are applied at an early stage of development with a higher degree of uncertainty compared with the time of accelerated assessment requests. The criteria target medicinal products of major public health interest, from the viewpoint of therapeutic innovation.^8^

Many PRIME designations also have OD, meaning that this scheme might have a positive impact on products addressing unmet medical needs. The EMA reports that, after 5 years of running the PRIME scheme, 56% of the 95 PRIME designations granted between 2016 and 2021 had a prior OD. Forty-four of the total PRIME designations were for ATMPs. The number is even higher at the time of MA, where 89% of the 18 PRIME designated products that received a MA had an OD. It should also be noted that 7 of the 18 products were ATMPs.^7^ This would indicate that applicants are using the two incentive programs closely. In the case of gene therapies for rare non-oncological conditions, however, this does not seem to be the case.

In this case, the COMP’s findings were that in non-oncological rare diseases where a gene therapy had received OD only 15% had applied for PRIME designation. This is an interesting finding as 66% of these designations were targeting conditions with a prevalence of less than 1 in 10,000. Many have a high unmet need, such as the retinopathies, where few if any products have a MA. High unmet medical need is one of the criteria for PRIME, although it should be noted that preliminary clinical evidence is also needed, and many of the ODs granted in this area only had non-clinical *in vivo* data indicating that they do not meet all the criteria for PRIME. In this respect, only 4 of the 95 PRIME-designated products entered the scheme through the early entry route, which is only available for SMEs and academia applicants and requires pharmacokinetic data in addition to non-clinical data.^7^ Since only 23% of the ODs in our analysis were granted to SMEs and academia collectively, and that most of the data provided for OD were non-clinical, the low number of PRIME designations for ODs for gene replacement therapies in non-oncological rare conditions is not surprising.

It should, however, be noted that, when looking at the 11 gene therapy products authorized in the EU, 7 were marketed during or after 2016 all of them having an OD, namely Zynteglo, Zolgesma, Libmeldy, Skysona, Upstanza, Roctavian, and Hemgenix, and 5 had both an OD and PRIME designations. This would indicate that use of the two incentives together can lead help to obtain a license with the caveat that the numbers to support this are very small.

In conclusion, the COMP noted that it has received an increasing number of submissions and granted designations for gene therapies in the period between 2016 and 2021 targeting non-oncological rare conditions. Many of these conditions had a prevalence of less than 1 in 10,000. There was no specific type of sponsor driving the submissions, indicating that the submissions come from a broad and varied base. Interestingly, academics and charities represented 16% of the submissions. Non-replicative viral vectors such as AAV vectors, as opposed to other types of viral vectors, were the most used in delivering the replacement gene. This concurs with what is reported in the literature regarding non-oncological conditions and the desired therapeutic aim. It was also noted that the most common conditions targeted were lysosomal disorders, eye disorders, neurological disorders, and hematological disorders. This finding seems to be at variance with what is reported in recent literature where neurological and eye disorders are the most common. Of particular interest was the report in the public domain that, in the top 10 conditions targeted by gene therapies, ALS was the ninth most common condition followed by retinitis pigmentosa, which was tenth.^13^ The conditions above were these were all oncological. The COMP noted that they had not granted ODs for gene replacement therapies for ALS.

The use of post-designation incentives such as PA still do not seem to be used sufficiently in an area where there is often a high unmet need. Medicinal development can be a challenge because of the low prevalence of less than 1 in 10,000, which itself is associated with small patient populations and limited recruitment for randomized clinical trials, thereby limiting data generation for regulatory purposes. PA can help to mitigate and potentially overcome clinical data generation concerns. The COMP also noted a low number of ODs for gene therapies in non-oncological conditions who were granted a PRIME designation. As noted earlier, many of these rare conditions have a high unmet need and thus should be eligible and benefit from this scheme. Of the seven market authorizations after 2016 which also obtained an OD, five also had a PRIME designation. Although the numbers are very small this would indicate that the two incentive programs could increase the possibilities of obtaining a license. An increase in awareness of those developing these products in these conditions about the benefits of the scheme could shorten the time to market and thus benefit patients who currently have no authorized treatments for their condition.

## Materials and methods

The COMP noted that, although several gene therapies were submitted between 2000 and 2016, the number was quite modest until around 2016, and it was hence decided to commence the investigation from that point in time. Cut-off date was defined as opinion from the COMP in December 2021. Summary assessment reports produced by the COMP at the time of assessing the OD submission were extracted from the corresponding EMA databases (DREAM and IRIS) using search words derived from the ATMP definitions (per Regulation (EC) No 1394/2007) such as ‘gene therapy,’ ‘viral vector-based,’ and ‘advanced therapy medicinal product.’ Cell therapy products, gene editing products, and tissue-engineered products were discarded in this step. Once the filters were applied (year of designation [2016–2021], type of product [viral vector based only] and non-oncological conditions), a total of 114 applications were retrieved and were subject of further analysis.

The data extraction from these reports was conducted by volunteers from the COMP and data cleaning was jointly done between COMP and EMA. Raw data were then analyzed under several categories. Those included administrative information on gene replacement therapies (outcome of OD application assessment), type of sponsor (large pharma, consultancy, SMEs, or academic and charity), orphan condition, prevalence of the sought OD condition, PA sought, and PRIME that were granted.

In addition, a detailed analysis of the type of viral vector backbone used and treatment modality (*in vivo* or *ex vivo*) were also conducted. AAV-derived products were also studied and classified according to their serotype over the years.

The Community Register of orphan medicinal products was accessed in November 2022 to investigate transfers between sponsors of the OD. When the international landscape of ODs was studied, the FDA and Japanese Ministry of Health, Labour and Welfare websites and the European Community Register of orphan medicines were accessed in July 2023.

All data are shown in the graphs as percentage or as number of applications from the total (n = 114).

## Data and code availability

Nothing to declare.
